# HIF1A Regulates *Rhbg* Expression to Enhance Ammonia Excretion in Amur Ide (*Leuciscus waleckii*) Under Extreme Alkaline Conditions

**DOI:** 10.3390/biology14050498

**Published:** 2025-05-02

**Authors:** Xuefei Zhao, Yu Zhang, Shuqi Li, Suying Bai, Wei Zhang, Yanchun Xu, Yumei Chang

**Affiliations:** 1 College of Wildlife and Protected Area, Northeast Forestry University, Harbin 150040, China; zhaoxuefei@nefu.edu.cn (X.Z.); zhangyunefu@163.com (Y.Z.); graceli@nefu.edu.cn (S.L.); sybai@nefu.edu.cn (S.B.); zwfur@nefu.edu.cn (W.Z.); 2 National Forestry and Grassland Administration Research Center of Engineering Technology for Wildlife Conservation and Utilization, Harbin 150040, China; 3 National and Local Joint Engineering Laboratory for Freshwater Fish Breeding, Heilongjiang River Fisheries Research Institute, Chinese Academy of Fishery Sciences, Harbin 150070, China

**Keywords:** ammonia excretion, *Leuciscus waleckii*, HIF1A, RHBG, alkali tolerant fish

## Abstract

High-alkalinity water bodies can disrupt normal ammonia metabolism in fish, leading to ammonia poisoning. In China, there exists a highly tolerant group of Amur ide (*Leuciscus waleckii*) that can survive in extreme alkaline lakes with alkalinity up to 53.57 mM (pH 9.6), making it an excellent model for elucidating the high-alkalinity tolerance mechanism in fish. We have discovered that this species has evolved a special ammonia excretion mechanism to maintain ammonia efflux in high-alkalinity environments. Compared to the freshwater forms of Amur ide, the ammonia excretion protein RHBG plays a prominent role in the ammonia excretion process of the alkali forms of Amur ide; however, the regulatory mechanism of RHBG expression in fish remains unclear. Through DNA pull-down, RNA-Seq, qPCR, Western blotting, immunofluorescence, and dual-luciferase reporter assays, this study demonstrates that the transcription factor HIF1A can inversely regulate the expression of *Rhbg* by binding to its promoter region, thereby participating in the high-alkalinity adaptation process of fish. The findings of this study provide a theoretical basis for elucidating the ammonia excretion mechanism and revealing the alkalinity tolerance mechanism in fish.

## Simple Summary

High-alkalinity aquatic environments can disrupt normal ammonia metabolism in fish, resulting in ammonia toxicity. Notably, *Leuciscus waleckii* exhibits remarkable tolerance to extreme alkalinity (54 mM, pH 9.6), serving as an ideal model for investigating the mechanisms underlying high-alkalinity tolerance in fish. Our findings suggest that the ammonia excretion protein RHBG may play a pivotal role in the restoration of ammonia metabolism in this species. However, the regulatory mechanisms governing RHBG expression in fish gills remain elusive. Employing a combination of techniques, including DNA pull-down, RNA-Seq, qPCR, Western blotting, immunofluorescence, and dual-luciferase reporter assays, we provide evidence that the transcription factor HIF1A can negatively regulate *Rhbg* expression by binding to its promoter region, thereby contributing to the adaptation of fish to high-alkalinity conditions. These results offer valuable theoretical insights into the mechanisms of ammonia excretion and alkalinity tolerance in fish.

## 1. Introduction

Globally, saline–alkali water bodies cover vast areas and continue to expand under the influence of human activities and climate change [1,2,3]. The effective development and utilization of these water bodies have attracted widespread attention from scholars [4,5]. Aquaculture is undoubtedly one of the most effective methods for this purpose [6,7]. However, due to the complex ionic composition and the imbalance in the content and proportions of some ions in saline–alkali water bodies, various abnormal physiological and biochemical reactions can occur in fish, threatening their homeostatic balance and making it difficult for most fish species to survive in such environments. This presents significant challenges for aquaculture practices. Currently, our understanding of the detailed mechanisms by which fish adapt to saline–alkali environments is still unclear, leading to the immaturity of cultivation methods for saline–alkali-tolerant fish. Therefore, there is a need for a deeper understanding of the molecular mechanisms of fish adaptation to saline–alkali conditions.

The Amur ide (*Leuciscus waleckii*), belonging to the Cyprinidae family, possesses unique amphibiotic adaptability to both fresh and alkaline waters. Populations inhabiting Dali Lake in Inner Mongolia, China, not only exhibit high economic value but also demonstrate tolerance to the rarely encountered extreme alkalinity of 53.57 mM (pH 9.6) in the lake [8]. The alkali form of Amur ide thrives in the lake and migrates to freshwater for reproduction. Our research has shown that, under long-term exposure to high alkalinity, the freshwater form of Amur ide exhibits a series of phenomena such as gill lamellae damage, cell fusion, and shedding. In contrast, the gill tissue of the alkali form of Amur ide remains structurally intact overall, with adaptations such as significantly widened gill filaments, elongated gill lamellae, and increased spacing between gill lamellae to accommodate the extreme external environment [9]. Despite their different adaptive capacities to high alkalinity, they belong to the same species, with a divergence time of less than 10,000 years, indicating they are in the initial stages of speciation. This makes them an ideal model for studying the mechanisms of fish adaptation to high-alkalinity environments [10]. Through comprehensive multi-omics research, we have discovered that the alkali form of Amur ide undergoes systematic adaptive evolution through acid–base regulation, osmoregulation, ion homeostasis, immune response, respiratory and circulatory system function, and other mechanisms to adapt to high-alkalinity water bodies [11,12,13,14]. Further verification has revealed that, compared to the freshwater form of Amur ide, the gill tissue of the alkali form of Amur ide possesses more robust regulatory capabilities, enabling it to maintain internal osmotic pressure and acid–base balance in high-alkalinity environments [15].

Maintaining normal ammonia metabolism is crucial for the growth and reproduction of fish. High concentrations of ammonia nitrogen can alter the ammonia metabolism of fish, damage their respiratory and immune systems, cause physiological dysfunction, increase their risk of infection, and ultimately lead to death [16,17,18]. Our recent research has found that the inability to effectively reduce the accumulation of ammonia in the body may be one of the important reasons why the freshwater form of Amur ide cannot adapt to extreme environments [19]. Ammonia is primarily excreted out of the body through concentration gradient-driven diffusion in the gills. High alkalinity directly disrupts the equilibrium between gaseous NH_3_ and NH_4_^+^ in water, narrowing the blood-to-water ammonia partial pressure gradient (Δ*P*_NH3_) across the gill epithelium and impeding the excretion of toxic ammonia [20,21,22].

In the ammonia excretion model of freshwater fish, NH_3_ is released from red blood cells by RHAG and then transferred to ionocytes by RHBG located on the basolateral membrane. It is then transported out of the body through RHCG in conjunction with V-type H^+^ ATPase, Na^+^/H^+^ exchanger proteins (NHE2/NHE3), and other membrane transport proteins, forming an excretion–metabolism complex that facilitates ammonia excretion [23]. We have uncovered a unique ammonia excretion mechanism evolved by the alkali form of Amur ide in high-alkalinity environments. This species can promote ammonia excretion and maintain homeostasis by upregulating the expression of ammonia transporters in the gills and through other mechanisms [19]. Among these, the ammonia excretion protein RHBG exhibits particularly notable expression, with its levels in the gills of the alkali form of Amur ide significantly upregulated after exposure to high-alkalinity conditions. We speculate that RHBG likely plays a crucial role in the ammonia excretion process during the adaptation of Amur ide to extreme environments [19]. However, the role of RHBG in ammonia excretion in fish has not been fully characterized [24], and the specific mechanism by which the alkali form of Amur ide upregulates RHBG expression remains unknown. Therefore, this study aims to delve into the regulatory mechanism of RHBG expression in the alkali form of Amur ide, which will provide a theoretical basis for further research into the mechanisms of high-alkalinity adaptation in fish.

## 2. Materials and Methods

### 2.1. Ethic Statements

This study was authorized by the Northeast Forestry University Science and Technology Ethics Committee (No:2024099).

### 2.2. Source of Fish Individuals and Their Management

The experimental fish were artificially bred offspring collected from Dali Lake. Before the experiment, 60 one-year-old pond-reared experimental fish were randomly selected and transferred to an indoor recirculating aquarium for a week of domestication. Water quality was monitored using a YSI water analyzer (YSI, Greene County, OH, USA), with water temperature maintained at 14.49 ± 0.45 °C, dissolved oxygen at 7.44 ± 0.45 mg/L, salinity at 0.06 ± 0.01‰, and pH value at 7.56 ± 0.09. The experimental fish had an average body weight of 121.57 ± 12.57 g and an average body length of 23.58 ± 0.88 cm. During the domestication period, the water in the aquarium was changed twice daily, and the fish were fed twice daily, with a 48-h fasting period before the experiment.

### 2.3. Bicarbonate AW Exposure and Fish Sampling

Alkaline water (AW) with an alkalinity of 50mM was prepared using sodium bicarbonate, and the experimental fish were subjected to a 7-day alkalinity stress treatment. Within this environment, 15 experimental fish were placed, with 3 replicate groups established, while a freshwater control group was concurrently set up. The water was changed once daily, with a 50% water exchange each time. The water temperature was monitored using a YSI water analyzer, maintained at 16.21 ± 0.45 °C, with dissolved oxygen at 7.39 ± 0.4 mg/L, salinity at 2.08 ± 0.12‰, pH ranging from 8.89 ± 0.16, and alkalinity at 49.96 ± 0.42 mM.

On days 1, 3, 5, and 7 after the stress treatment, 3 fish were randomly selected from each replicate group, totaling 9 fish for tissue collection. For the control group, 3 samples were randomly collected on days 1, 3, and 7, respectively, and labeled as 0d. All fish were anesthetized with 100 mg/L of neutralized MS222 (Pharmaq Ltd., Hampshire, UK). The left gill of each experimental fish was removed and fixed in a 4% paraformaldehyde solution for immunofluorescence staining. Simultaneously, the right gill tissues from each individual were collected, frozen immediately in liquid nitrogen, and stored at −80 °C for subsequent experimental analysis.

### 2.4. DNA Pull-Down and LC-MS

The NE-PER Nuclear and Cytoplasmic Extraction Reagents (Thermo Fisher Scientific, 78833, Waltham, MA, USA) were used for the separation of nuclear proteins from gill tissue in the 7-day experimental group. The BCA method was employed for protein concentration determination, while the DNA pull-down Kit for Animals (Sangon Biotech, B605112, Shanghai, China) was utilized for DNA pull-down assays. Probes targeting the promoter region of the *Rhbg* gene were labeled with desthiobiotin, and unlabeled control probes were also set up. In the experiment, labeled and unlabeled probes were incubated with nuclear proteins, respectively, while a control group without probes was also included. The protocols and procedures followed the instructions provided in the kit manuals. SDS-PAGE electrophoresis was performed to assess the binding of specific proteins to the probes.

After electrophoresis, the protein bands were excised from the gel and washed with water. The gel pieces were then decolorized with 50% MeOH/50 mM NH_4_HCO_3_ at 37 °C for 30 min. The supernatant was removed, and the gel was dehydrated by adding 100% ACN and shaking. Dried gel particles were treated with 25 mM DTT/50 mM NH_4_HCO_3_ at 56 °C for 30 min. After removing DTT, 55 mM IAA/50 mM NH_4_HCO_3_ was added, and the reaction was allowed to proceed in the dark at room temperature for 30 min. The supernatant was discarded, and the gel particles were washed with water three times and dehydrated with 100% ACN until they turned white. Trypsin, diluted to a final concentration of 20 ng/µL with 25 mM NH_4_HCO_3_, was added to the gel particles, and the mixture was incubated on ice for 30 min. An additional 25 mM NH_4_HCO_3_ was added, and the gel particles were digested overnight at 37 °C in a constant temperature incubator. The next day, the samples were sonicated for 15–20 min in an ultrasonic bath to extract the peptides. The peptides were purified using ZipTip and then analyzed and identified by liquid chromatography–mass spectrometry (LC-MS) using a Dionex Ultimate^TM^ 3000 RSLCnano System (Thermo Fisher Scientific, Waltham, MA, USA) and the quadrupole Orbitrap mass spectrometer (Q Exactive, Thermo Fisher Scientific, MA, USA) to obtain the target proteins. The raw mass spectrometry files were processed and converted into MGF format using MM File Conversion software (https://mm-file-conversion.software.informer.com/3.9/#google_vignette accessed on 10 March 2024) and then searched against the UniProt database using MASCOT (http://www.matrixscience.com/ accessed on 10 March 2024).

### 2.5. RNA-Seq

In previous studies, we observed that the expression level of *Rhbg* was significantly upregulated starting from the third day of exposure to 50 mM alkalinity stress [25]. Therefore, we conducted transcriptome sequencing on gill tissues collected on the third day of treatment, along with a control group, in order to jointly screen for target transcription factors involved in regulating *Rhbg* expression using DNA pull-down assays.

Three gill tissue samples from both the 3d and 0d time points, with three samples from each, were randomly selected for library construction and sequencing on the Illumina high-throughput sequencing platform. This process was outsourced to Unitedomics Biotech Co., Ltd (Tianjin, China). A brief overview of the analytical workflow is as follows: The NEBNext^®^ Ultra™ RNA Library Prep Kit for Illumina^®^ (NEB, Ipswich, MA, USA, E7530) was used for library preparation. Qualified sequencing libraries were sequenced on the Illumina NovaSeq6000, San Diego, CA, USA sequencing platform in PE150 mode. The raw results were converted into Raw Data in fastq format using bcl2fastq software (v2.20) and further processed to remove reads containing adapters and low-quality reads, resulting in Clean Reads for subsequent analysis. Clean Reads were aligned with the reference genome using HISAT2 (v2.2.0) software to obtain the positional information of the reads on the reference genome. The expression levels of genes/transcripts were normalized using FPKM or TPM. Multiple testing correction was performed using the Benjamini–Hochberg method. Differential expression analysis of transcripts was conducted using DESeq2. Genes were deemed significantly differentially expressed based on the criteria of padj ≤ 0.05 and log2 Fold change ≥ 1. InterProScan was used to analyze the GO (Gene Ontology) results of unigenes with consolidated databases of InterPro. Subsequently, KOBAS was employed to obtain the KEGG (Kyoto Encyclopedia of Genes and Genomes) results of unigenes within the KEGG pathway. After predicting the amino acid sequences of the unigenes, (HMMER software http://hmmer.org/ accessed on 10 March 2024) was used to align these sequences against the Pfam database, thereby acquiring annotation information for the unigenes.

### 2.6. Phylogenetic Analysis

The *Hif1α* (Hypoxia-inducible Factor 1-alpha) gene sequence was obtained from the database of Amur ide and converted into its amino acid sequence. By comparing it with HIF1A amino acid sequences from species that have high genetic relationships with Amur ide, which were collected from NCBI GenBank, a multiple sequence alignment was conducted using MEGA7.0 [26]. An evolutionary tree based on the Kimura 2-parameter (K2P) model was constructed using the Neighbor–Joining method. This tree was subjected to 1000 bootstrap replicates for robustness. The HIF1A amino acid sequence of Atlantic salmon (*Salmo salar*, NP_001133494.1) was chosen as the outgroup for the phylogenetic analysis.

### 2.7. Quantitative Real-Time PCR (qPCR)

Each set of three gill tissues was mixed to form a single sample (*n* = 3, totaling nine samples). The FastPure Cell/Tissue Total RNA Isolation Kit V2 (Vazyme, RC112-01, Nanjing, China) was used for total RNA purification, following the instructions provided in the kit. The NanoDrop 8000 spectrophotometer (Thermo Fisher Scientific, MA, USA) was employed to assess the quality and concentration of the RNA.

For the synthesis of the first strand of cDNA, the PrimeScript™ RT Reagent Kit with gDNA Eraser (Takara, Kyoto, Japan) was utilized, following the reaction system and steps provided in the kit instructions. qPCR detection was performed using SYBR Premix Ex Taq™ II (Takara, Kyoto, Japan) and the CFX384 Touch Real-Time PCR System (Bio-Rad, Hercules, CA, USA), with the reaction system and program based on the kit instructions. Melting curve analysis was conducted using the default program of the instrument. *β-actin* was used as the internal reference gene for result normalization, and data analysis was completed using the 2^-ΔΔCt^ method. Detailed primer information is provided in Table 1.

### 2.8. Western Blotting

A total of 100 mg of gill tissue collected from both 0d and 3d stages (*n* = 3, with each group of 3 samples mixed into 1, totaling 9 samples) was thoroughly ground under liquid nitrogen. After adding 250 μL of RIPA lysis buffer (Beyotime, Beijing, China), the mixture was transferred to a vortex mixer and mixed at the highest speed until fully homogenized. The sample was then incubated on ice for 10 min, with multiple mixing intervals to ensure complete lysis. Using a refrigerated centrifuge, the sample was centrifuged at 12,000 rpm for 10 min at 4 °C. The supernatant was collected and transferred to a new centrifuge tube, representing the total protein of the sample.

The BCA method (Beyotime, Beijing, China) was used to determine the protein concentration. After adjusting the protein loading volume, 4× Protein SDS-PAGE Loading Buffer (Takara, 9173, Kyoto, Japan) was added and thoroughly mixed. The mixture was incubated at 99 °C for 10 min to ensure protein denaturation. A 4–20% gradient SDS-PAGE gel (Genscript, M00655, Nanjing, China) was used for protein separation under electrophoresis conditions of 120V for 50 min.

After electrophoresis, the proteins were transferred onto a PVDF membrane under transfer conditions of 200 mA for 1 h. Following the transfer, the membrane was blocked with 5% BSA at room temperature for 1 h and then incubated with the primary antibody (rabbit anti-RHBG antibody prepared in previous studies for Amur ide, 50 KD; HIF1A, MCE, HY-P80704, 93 KD, Kenilworth, NJ, USA; ACTB, Proteintech, 66009-1-Ig, 42 KD, Chicago, IL, USA) overnight at 4 °C. The next day, after washing several times with TBST buffer, the membrane was incubated with the secondary antibody (Proteintech, SA00001-1 or Proteintech, SA00001-2, Chicago, IL, USA) at room temperature for 1 h. After another wash with TBST buffer, the ECL method was used for visualization. The Bio-Rad ChemiDoc MP Imaging System was employed for image acquisition, and ImageJ software version 1.8.0 was used for gray-scale analysis of the bands.

### 2.9. Cryosections and Immunofluorescence Staining

Before cryosectioning, gill tissues from 0d and 3d were fixed in 4% paraformaldehyde overnight and then transferred to MeOH. They were subsequently rehydrated step-by-step for 10 min through a MeOH:PBS series (3:1; 1:1; 1:3) and 1 × PBS. The samples were then rinsed three times with PBS and dehydrated for 1 h each in 5%, 10%, and 20% sucrose/PBS (*w*/*v*), respectively, before being embedded in Tissue-Tek O.C.T. (Tissue Tek, Torrance, CA, USA). Sections with a thickness of 10 μm were prepared using a freezing microtome (Leica CM3050 S, Solms, Wentzler, Germany).

For immunofluorescence experiments, the slides were washed with 1 × PBS for 20 min and blocked with 5% BSA at room temperature for 1 h. Thereafter, the sections were incubated overnight at 4 °C with rabbit polyclonal antibodies against RHBG of the Amur ide and HIF1A (MCE, Kenilworth, NJ, USA, diluted 1:200). The next day, after washing three times with 1 × PBS, the sections were incubated with fluorescein isothiocyanate (FITC) 488-conjugated goat anti-rabbit IgG (diluted 1:200) for 1 h at room temperature. Fluorescent signals and images were acquired using an inverted fluorescence microscope (Zeiss Axio Observer 3, Oberkochen, Germany).

### 2.10. Dual-Luciferase Reporter Assay

The CDS region of the *Hif1α* gene and the promoter region of the *Rhbg* gene were constructed into the pCDNA3.1 and pGL3-basic vectors, respectively. After completing the molecular cloning, plasmid extraction was performed. The constructed reporter plasmid, overexpression plasmid, and empty vector were co-transfected into HEK293T cells. Three experimental groups were set up as follows: (1) the group with empty plasmids (pCDNA3.1 and pGL3-basic); (2) the group with the empty plasmid (pCDNA3.1) and the pGL3-basic-*Rhbg* promoter reporter plasmid; (3) the group with the pCDNA3.1-*Hif1α* overexpression plasmid and the pGL3-basic-*Rhbg* promoter reporter plasmid. The Dual-Luciferase^®^ Reporter Assay System (Promega, E1910, Madison, WI, USA) and the FlexStation3 Multi-Mode Microplate Reader were used for result detection, following the system configuration and steps outlined in the kit instructions.

### 2.11. Statistical Analysis

Statistical significance was assessed using one-way ANOVA and Student’s *t*-test. All statistical analyses were conducted using GraphPad Prism 9.5.1 software, and the results are presented as mean ± standard deviation (SD). *p*-value < 0.05 was considered significant.

## 3. Results

### 3.1. The Proteins Binding to the Promoter Region of Rhbg Were Identified Through DNA Pull-Down and LC-MS

**Figure 1:**
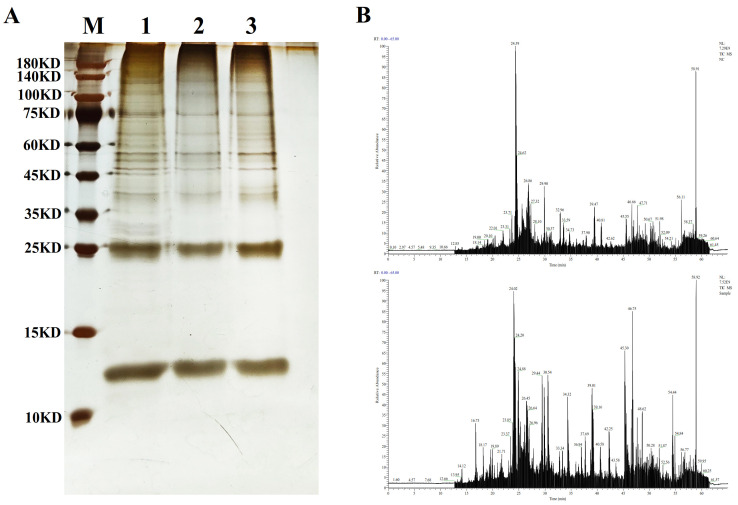
(**A**) SDS–PAGE gel diagram of DNA pull-down. M: protein marker. Lane 1: labeled probe+Bioeast Mag-SA+protein. Lane 2: unlabeled probe+Bioeast Mag-SA+protein. Lane 3: Bioeast Mag-SA+protein. (**B**) The total ion chromatogram of the unlabeled control group and the labeled probe group.

To obtain proteins that may interact with the *Rhbg* promoter region, we employed a DNA pull-down method combined with LC-MS for screening. Nuclear proteins were extracted from gill tissues treated with high alkalinity for 7 days and analyzed using DNA pull-down, followed by separation of the products via SDS-PAGE. As shown in Figure 1A, lane 2 represents the control group, where we observed a significant reduction in the amount of proteins bound by the unlabeled probe compared to the labeled probe group in lane 1, indicating specific binding of the desthiobiotin-labeled probe group to the proteins. Lane 3 serves as a no-probe control, where the amount of non-specifically bound proteins to the Bioeast Mag-SA beads is also low, suggesting reliable experimental results.

Upon comparison, we found visible differential bands between the different groups. The pull-down products of the desthiobiotin-labeled DNA probe and the unlabeled control group were excised from the gel for protease digestion and peptide purification. The peptide samples were separated by liquid chromatography and detected by mass spectrometry to screen for differentially expressed proteins. In Figure 1B, the total ion chromatogram of the unlabeled control group exhibits significant differences from that of the desthiobiotin-labeled probe group.

### 3.2. RNA-seq Results of Gill Tissues Under Different Alkalinity Treatment Durations

**Figure 2:**
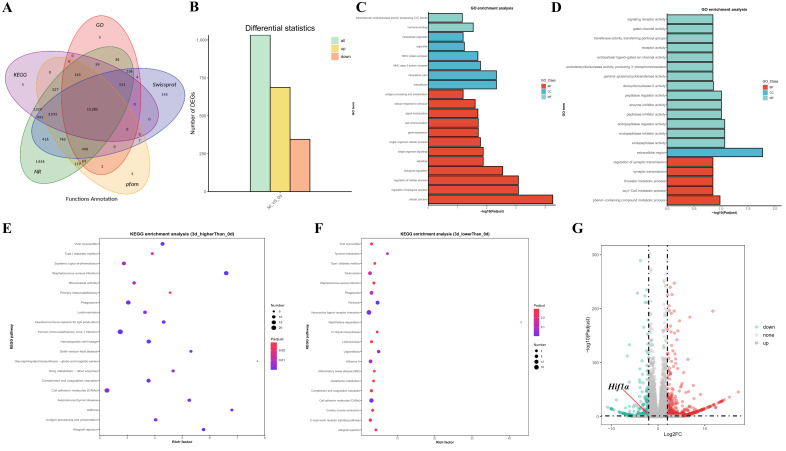
(**A**) Venn diagram for functions annotation; (**B**) Statistical graph of differentially expressed transcripts; (**C**) GO classification statistical graph of transcripts with higher expression levels in 3d compared to 0d; (**D**) GO classification statistical graph of transcripts with lower expression levels in 3d compared to 0d; (**E**) KEGG classification statistical graph of transcripts with higher expression levels in 3d compared to 0d; (**F**) KEGG classification statistical graph of transcripts with lower expression levels in 3d compared to 0d; (**G**) Volcano plot of differentially expressed transcripts.

The transcripts obtained through sequencing were aligned with databases including NR, Swiss-Prot, COG, KOG, eggNOG4.5, and KEGG. The results are shown in Figure 2A, with a total of 15,285 transcripts successfully aligned across these databases. When comparing gill tissues from the 3d group with those from the 0d group, a total of 1032 differentially expressed genes (DEGs) were identified, including 687 upregulated genes and 345 downregulated genes (Figure 2B). GO enrichment analysis revealed that, compared to the 0d control group, the upregulated genes at 3d were significantly enriched in terms such as cellular process, regulation of biological process, regulation of cellular process, and biological regulation (Figure 2C), while the downregulated genes were significantly enriched in terms like gated channel activity, extracellular ligand-gated ion channel activity, and phenol-containing compound metabolic process (Figure 2D). The significant differential expression of these genes suggests that the fish initiates an immune response, activates ion channels for ion regulation, and regulates metabolic and other biological processes to respond to changes in the external environment. In the KEGG enrichment analysis results, the upregulated genes were significantly enriched in pathways such as Glycosphingolipid biosynthesis-globo and isoglobo series and cell adhesion molecules (Figure 2E), while the downregulated genes were significantly enriched in pathways including glutathione metabolism, cell adhesion molecules, and N-glycan biosynthesis (Figure 2F), suggesting that multiple biological processes are involved in the fish’s adaptation to high alkalinity. By combining transcriptome sequencing with DNA pull-down sequencing results, we initially screened out the target transcription factor HIF1A (Figure 2G).

### 3.3. Analysis of the Hif1α Gene Sequence in the Alkali Form of Amur Ide

**Figure 3:**
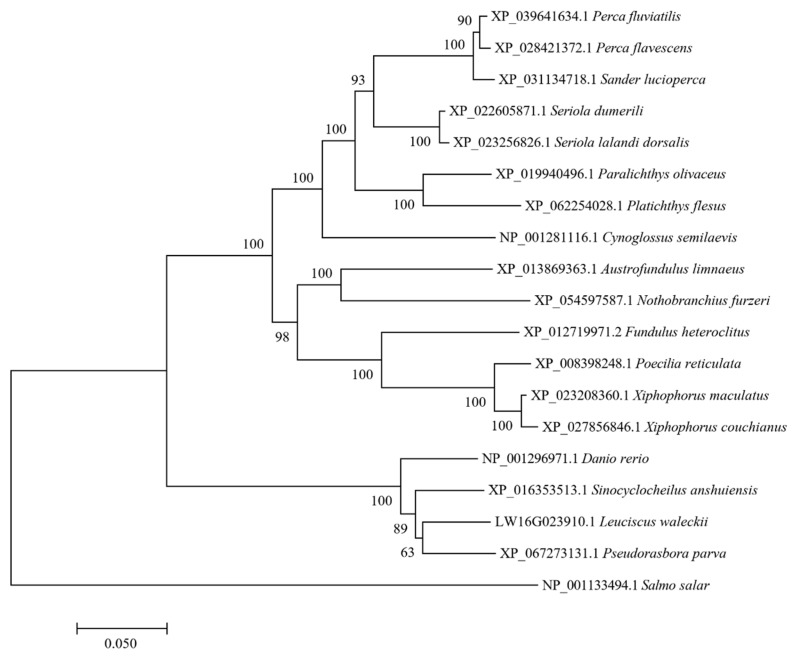
NJ phylogenetic tree constructed using the amino acid sequence of the *Hif1α* gene from different fish species.

We obtained the *Hif1α* gene sequence from the Amur ide genome database. Analysis revealed that the gene has a total length of 3956 bp, encoding 774 amino acids. Through a comprehensive search of homologous databases, we selected 18 fish HIF1A amino acid sequences and constructed a phylogenetic tree using the Neighbor–Joining Method in MEGA 7.0 software. As shown in Figure 3, the 18 species were clustered into respective orders such as Perciformes, Pleuronectiformes, and Cypriniformes, and they also mostly grouped with species from the same family. Specifically, Amur ide LW16G023910.1 clustered with carp family fish such as zebrafish (*Danio rerio*).

### 3.4. Impact of High Alkalinity on HIF1A Expression

**Figure 4:**
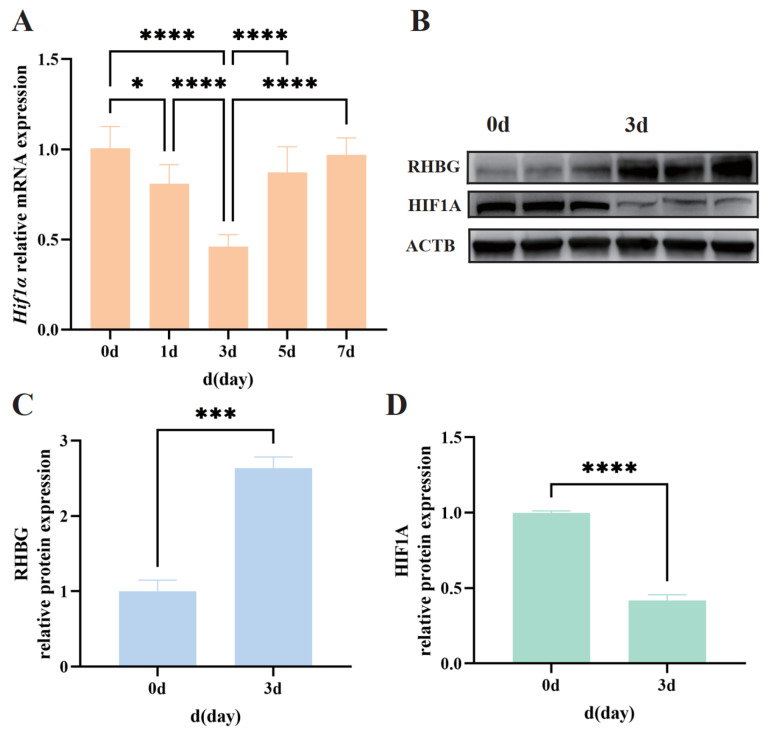
Changes in expression levels of HIF1A and RHBG in gills of Amur ide under 50 mM alkalinity treatment. * *p* < 0.05, *** 0.0001 < *p* < 0.001, **** *p* < 0.0001. (**A**) Relative mRNA expression levels of *Hif1α* after 7-day alkaline treatment; (**B**) Protein expression levels of HIF1A, RHBG, and ACTB under alkaline treatment of 0d and 3d; (**C**) Relative protein expression levels of RHBG under alkaline treatment of 0d and 3d; (**D**) Relative protein expression levels of HIF1A under alkaline treatment of 0d and 3d. Data are expressed as means ± SD (*n* = 3).

To investigate the impact of high alkalinity on the expression of the *Hif1α* gene, we examined the changes in *Hif1α* gene expression in the gills of the alkali form of Amur ide. During the seven-day alkalinity treatment, the mRNA expression levels of *Hif1α* in the gills showed an overall downregulation to varying degrees (Figure 4A). Notably, the downregulation was most significant at 3 days, with a decrease of more than half compared to the control group, and there were significant differences compared to other groups (*p* < 0.05). This is similar to our previous findings regarding the mRNA expression levels of *Rhbg* in the gills of the alkali form of Amur ide exposed to alkaline conditions, which also showed significant changes at 3 days to the control group [19].

**Figure 5:**
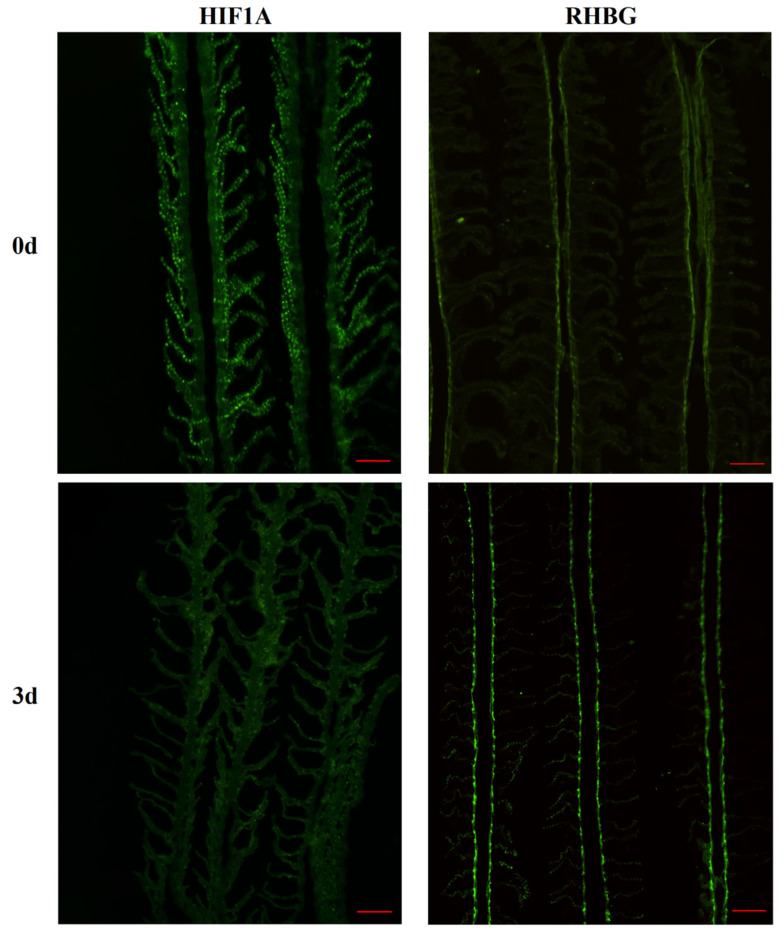
Expression of HIF1A and RHBG in the gills of Amur ide at 0d and 3d after treatment with 50 mM alkalinity. Scale bars are 100 μm.

Therefore, we further examined the protein expression of HIF1A and RHBG in the gills before and after high alkalinity treatment. The results showed that the protein expression levels were similar to the changes in mRNA expression, with a significant decrease in HIF1A protein expression at 3 days compared to the control group, while the protein expression of RHBG significantly increased at 3 days (Figure 4B). Grayscale value calculations revealed that the relative grayscale value of RHBG at 3 days was nearly three times that at 0 days, while the relative grayscale value of HIF1A at 3 days showed a similar decrease to the mRNA changes, with a reduction of more than half (Figure 4C,D; *p* < 0.05). We also verified these results in gill tissue using immunofluorescence techniques. As shown in Figure 5, compared to 0 days, the overall green fluorescence intensity of HIF1A in the gills was significantly weakened at 3 days, while the green fluorescence intensity of RHBG was significantly enhanced at 3 days. The mutual verification among the above results indicates that there may be a correlation between the expression changes of the two proteins after three days of alkaline treatment. The original WB figures can be found in Supplementary Material.

### 3.5. HIF1A Regulates the Expression of Rhbg

**Figure 6:**
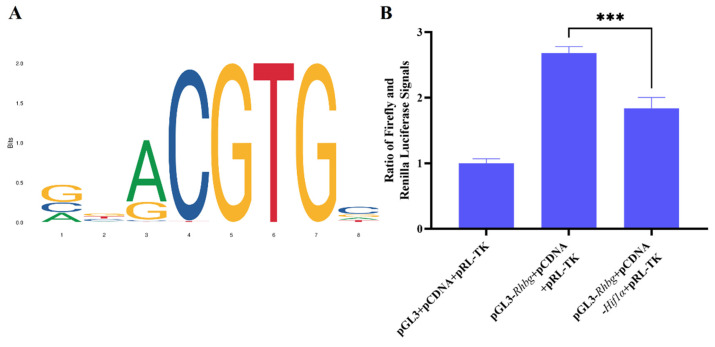
(**A**) The motif sequence of *Hif1α* obtained from the JASPAR database; (**B**) Dual-luciferase reporter assay verifying that HIF1A can bind to the *Rhbg* promoter. Data are expressed as means ± SD (*n* = 3). The asterisk (***) denotes a significant difference.

The DNA pull-down assay has confirmed the binding interaction between HIF1A and RHBG. Furthermore, we obtained the motif sequence of *Hif1α* from the JASPAR database (Figure 6A) and compared it with the promoter region of *Rhbg*. The presence of the motif sequence in the *Rhbg* promoter further substantiated the potential for binding between the two. This indicates that HIF1A can bind to the *Rhbg* promoter region, thereby exerting regulatory effects on *Rhbg* expression; however, it remains unclear whether this regulatory effect is positive or negative. The results of qPCR, WB, and IF all suggest opposite trends in the expression levels of HIF1A and RHBG at 50mM alkalinity, leading us to speculate that HIF1A may negatively regulate *Rhbg* expression by binding to the *Rhbg* promoter region.

To validate this hypothesis, we conducted a dual-luciferase reporter assay. Three groups were established for transfection: the pCDNA3.1 and pGL3-basic empty plasmid group, the pCDNA3.1 empty plasmid and pGL3-basic-*Rhbg* promoter reporter plasmid group, and the pCDNA3.1-*Hif1α* overexpression plasmid and pGL3-basic-*Rhbg* promoter reporter plasmid group. The total amount of plasmid transfected was equal across all groups. We found that the signal intensity in the group with additional transfection of the pCDNA3.1-*Hif1α* overexpression plasmid was significantly lower compared to the group only transfected with the pGL3-basic-*Rhbg* reporter plasmid, with a statistically significant difference between the two (Figure 6B, *p* < 0.05). This demonstrates that HIF1A can indeed bind to the *Rhbg* promoter and inhibit the expression of *Rhbg*.

## 4. Discussion

The impact of high-alkalinity environments on fish is complex, and the adaptive mechanisms of fish to such environments are not monolithic either. In this study, we observed from transcriptome sequencing results that various aspects of fish physiology were significantly affected after exposure to high alkalinity, including the regulation of ion channels, cellular electrophysiological activities or signal transduction, and multiple metabolic pathways. These findings are similar to those from our previous sequencing results after a seven-day alkalinity treatment [11,12,13,14]. Additionally, our GO and KEGG enrichment analyses revealed significant enrichment of numerous pathways related to immune responses. This is likely due to the initiation of immune responses in fish as a stress response to high alkalinity. Our ongoing research on blood physiology has found a marked enhancement of immune responses in fish after high-alkalinity treatment, which corroborates the findings of this study (unpublished). Maintaining normal ammonia excretion is crucial for fish in this process. Therefore, evolving specialized ammonia excretion mechanisms in high-alkalinity environments is a key component of fish alkalinity adaptation mechanisms.

Most fish primarily rely on the blood-to-water ammonia partial pressure gradient (Δ*P*_NH3_) across the gill epithelia to eliminate ammonia (85%) by diffusion [20]. High-alkaline environments disrupt this balance and impair normal ammonia excretion. Fish living in high-alkaline waters have evolved different ammonia excretion strategies to adapt to these extreme conditions, such as increasing tissue tolerance to ammonia [27,28], re-establishing Δ*P*_NH3_ [27,28,29], converting toxic ammonia into non-toxic urea-nitrogen [30,31], and elevating the expression levels of Rh proteins [32,33]. We found that the alkali form of Amur ide does not rely on a single strategy to cope with the impact of high-alkaline environments on ammonia excretion. When ammonia excretion is impeded due to high-alkaline stress, it converts ammonia into urea–nitrogen to reduce the risk of ammonia poisoning. Compared to its freshwater form, it exhibits greater ammonia tolerance, enabling it to maintain relative homeostasis in extreme environments and ultimately actively restore ammonia excretion by increasing the expression levels of Rh proteins and other related proteins [19].

During this process, the freshwater form of Amur ide still primarily relies on RHAG to attempt ammonia excretion, but the traditional ammonia excretion model for freshwater fish does not help it effectively restore ammonia excretion [19,23]. In contrast, the alkali form of Amur ide seems to rely more on RHBG, with a significant upregulation of RHBG levels in the gills, far exceeding those in the freshwater form. Braun et al. previously found RHBG expression in the blood vessels of the gills of blind eels after exposure to high ammonia environments and speculated, based on its unique localization, that RHBG likely replaces RHAG in ammonia transport from the blood to the gills [34]. Zimmer et al. discovered that RHBG in zebrafish can compensate for the loss of RHCG2 and exhibits stronger ammonia excretion capabilities [24]. Therefore, we believe that RHBG likely plays a crucial role in ammonia transfer during the restoration of ammonia excretion in the alkali form of Amur ide.

However, the regulation of RHBG expression in fish remains unclear. To further elucidate the detailed mechanisms underlying the specific regulation of RHBG expression in the alkali form of Amur ide, we used DNA pull-down to screen for proteins that may interact with the *Rhbg* promoter region and identified the transcription factor HIF1A. Additionally, by constructing a phylogenetic tree, we demonstrated that the amino acid sequence of HIF1A in the alkali form of the Amur ide still clusters with cyprinid fish. As one of the subunits of HIF1, HIF1A participates in regulating various processes such as cellular metabolism, immune responses, anti-infection, and defense [35,36,37], making it an important transcription factor. The expression of HIF1A is not only regulated by oxygen levels but also by various signaling pathways such as PI3K/AKT/mTOR and MAPK [38]. There are also significant species-specific differences in its expression among different fish. For example, the expression level of HIF1A protein in the gills of the *Fundulus grandis* is not affected by hypoxia [39]. In hypoxia-sensitive fish such as Blunt Snout Bream (*Megalobrama amblycephala*) and Rainbow Trout (*Oncorhynchus mykiss*), no significant changes in *Hif1α* expression were observed under hypoxic stress [25,40].

In our previous studies, among the genes belonging to the HIF family, *Hif-3a* exhibited a significant increase in expression levels during 24-h tolerance in the freshwater form of Amur ide, whereas it was significantly downregulated in the gills of the alkali form of Amur ide at 1 h, 3 h, 8 h, and 24 h post-alkalinity treatment [41]. In this study, we demonstrated a significant decrease in HIF1A expression levels after alkaline treatment by examining its mRNA transcription levels and protein expression levels. We previously measured the oxygen consumption rate of the fish during a 7-day period of high-alkalinity stress and found that the oxygen consumption rate of the alkali form of Amur ide fluctuated minimally overall, remaining at a level similar to that of the control group. There was no situation where alkalinity stress led to oxygen deficiency, necessitating an increase in oxygen consumption rate [19]. Therefore, changes in HIF1A expression in the alkali form of Amur ide can basically be ruled out as being influenced by oxygen content. We believe that this change is correlated with alterations in RHBG expression levels. This was also confirmed in the dual-luciferase reporter assay, which demonstrated that HIF1A can indeed bind to the promoter region of *Rhbg* and negatively regulate the expression of RHBG.

We hypothesize that under high-alkalinity stress, the alkaline form of Amur ide may initiate a series of response mechanisms to cope with the external extreme environment, including immune responses and defense mechanisms. During this process, it is possible that the expression level of HIF1A in the alkaline form of Amur ide is affected, leading to its downregulation, which in turn promotes the upregulation of RHBG expression. This regulatory mechanism helps to restore ammonia excretion in the gills, thereby assisting the organism in adapting to the external environment. Of course, this is likely a complex regulatory process, and there may be other transcription factors or transcriptional coregulators that are also playing roles. Much remains unclear to us in this regard, and more in-depth and extensive research is needed to uncover further details. In summary, this discovery not only deepens our understanding of the alkalinity tolerance mechanisms in fish but also aids in uncovering the unique ammonia excretion regulatory strategies evolved by fish to adapt to diverse environments. Furthermore, it provides a solid scientific foundation for further analyzing the adaptive evolution mechanisms of fish. This finding also offers new research directions for the cultivation of economically important fish species and the development of high-quality aquaculture strategies, significantly promoting the in-depth exploration of ammonia excretion mechanisms in fish. Additionally, it provides a breeding strategy for developing new varieties of alkaline-tolerant fish and enhancing the utilization efficiency of saline–alkaline water bodies.

## 5. Conclusions

This study revealed that the Amur ide enhances its ammonia excretion capability under extreme alkaline conditions by inversely regulating the expression of the ammonia excretion protein RHBG through the transcription factor HIF1A, thereby facilitating its adaptation to high-alkalinity environments. This finding provides a novel theoretical basis for understanding the mechanisms of high-alkalinity tolerance in fish.

## Figures and Tables

**Table 1:** Primer information for quantitative real-time PCR (qPCR).

Gene Names	Primer Sequences (5′-3′)	Sizes of Product (bp)
*Hif1α*	F: TCACCTCACCAAGACGCATC R: TCTGTCATGGTCTGCTGCAG	225
*β-actin*	F: AGCAGGAGTACGACGAGTCT R: ATCCTGAGTCAAGCGCCAAA	154

## Data Availability

The original contributions presented in this study are included in the article/Supplementary Material, and further inquiries can be directed to the corresponding author (s).

## Supplementary Materials

The following supporting information can be downloaded at: https://www.mdpi.com/article/10.3390/biology14050498/s1, Supplementary Material.
