# Supplementary figures and images for: HIF1A Regulates Rhbg Expression to Enhance Ammonia Excretion in Amur Ide (Leuciscus waleckii) Under Extreme Alkaline Conditions
Source: Biology (Basel). 2025 May 2;14(5):498. doi: 10.3390/biology14050498 (PMC12108939; doi:10.3390/biology14050498)

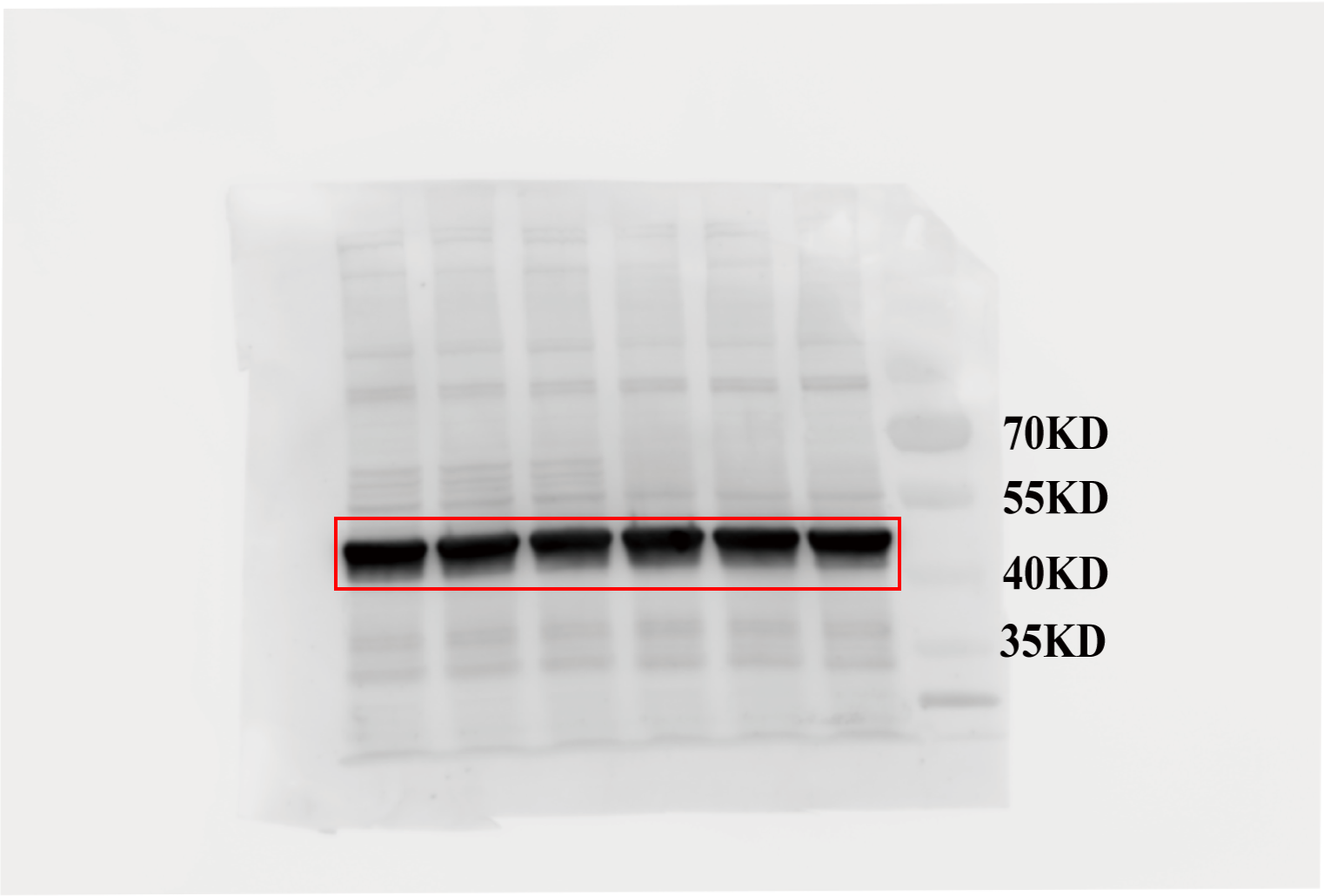

Supplement: Supplementary file 1 [file biology-14-00498-s001.zip › biology-3512273-supplementary/Uncropped_images/Figure 4-ACTB.tif]

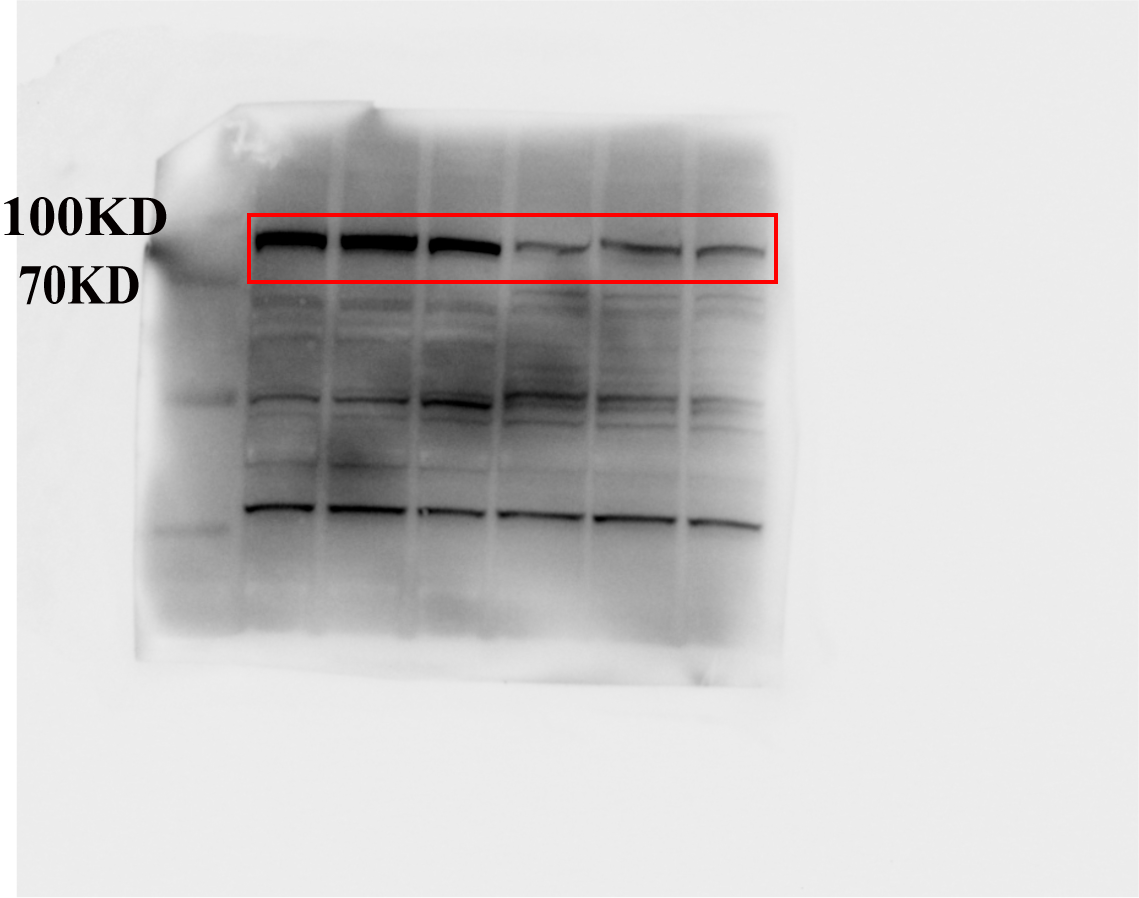

Supplement: Supplementary file 1 [file biology-14-00498-s001.zip › biology-3512273-supplementary/Uncropped_images/Figure 4-HIF1A.tif]

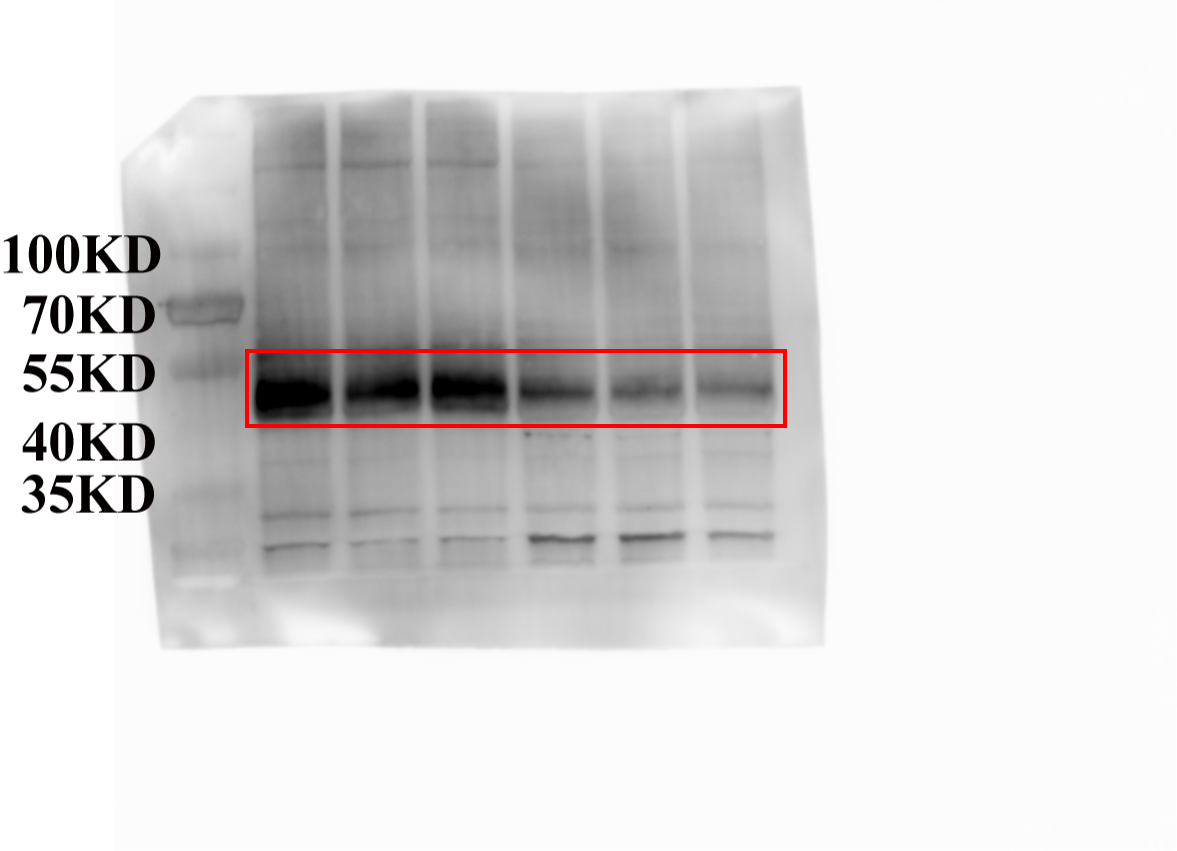

Supplement: Supplementary file 1 [file biology-14-00498-s001.zip › biology-3512273-supplementary/Uncropped_images/Figure 4-RHBG.tif]
